# Deceptive Techniques to Hide a Compressed Video Stream for Information Security

**DOI:** 10.3390/s21217200

**Published:** 2021-10-29

**Authors:** Jeonghwan Heo, Jechang Jeong

**Affiliations:** Department of Electronic Engineering, Hanyang University, Seoul 04763, Korea; hur881122@hanyang.ac.kr

**Keywords:** codec, information security, video encryption, deceptive techniques, high efficiency video coding, H.264/AVC, H.263, IVC

## Abstract

With the recent development of video compression methods, video transmission on traditional devices and video distribution using networks has increased in various devices such as drones, IP cameras, and small IoT devices. As a result, the demand for encryption techniques such as MPEG-DASH for transmitting streams over networks is increasing. These video stream security methods guarantee stream confidentiality. However, they do not hide the fact that the encrypted stream is being transmitted over the network. Considering that sniffing attacks can analyze the entropy of the stream and scan huge amounts of traffic on the network, to solve this problem, the deception method is required, which appears unencrypted but a confidential stream. In this paper, we propose the new deception method that utilizes standard NAL unit rules of video codec, where the unpromised device shows the cover video and the promised device shows the secret video for deceptive security. This method allows a low encryption cost and the stream to dodge entropy-based sniffing scan attacks. The proposed stream shows that successful decoding using five standard decoders and processing performance was 61% faster than the conventional encryption method in the test signal conformance set. In addition, a network encrypted stream scan method the HEDGE showed classification results that our stream is similar to a compressed video.

## 1. Introduction

Currently, with the development of high-efficiency video codecs (HEVC), video traffic is used more often, accounting for approximately 80% of the total network traffic [1]. Therefore, symmetric key encryption methods have been developed for the information security of video streams [2], and digital rights management (DRM) methods that transmit keys using encryption protocols with asymmetric keys are mainstream today [3]. The world wide web consortium (W3C) recommends that the stream be transmitted by encrypting the video stream through a combination of asymmetric key and symmetric key encryption in HTML5. These protocols are easy to apply to video encoders designed without encryption; therefore, it is spotlighted as a technique that implements DRM for content-streaming platforms. However, there are some problems with this technique of encrypting the entire stream. First, the encryption computational cost was high, meaning that it is difficult to miniaturize the device for use by IP cameras, drones, and IoT devices, and power consumption increases, which in turn makes mobile implementation difficult. Second, encryption is easily tracked by traffic eavesdropping techniques [4]. Cryptography provides nearly perfect mathematical confidentiality, but the high entropy of the byte stream makes it easy to expose in statistical analysis. Since the scope of data encryption is focused on stream datas, it is difficult to hide the sender and receiver on the network. Therefore, it is necessary to secure information by deceiving methods that prevent other people from inferring the contents through a wide range of network scan. Therefore, our motivation came from the need for secret video to prevent others from inferring the importance of the data, while undetected encryption.

Deception methods play an important role, especially within small networks, such as wireless LAN. This is because in a small network, it is easy to analyze the data of all the hosts to which they belong through the router to physically find the location of the monitoring assets hidden within a short distance. Given that the eavesdropping method through LAN sniffing uses the entropy analysis of the stream, it is possible to monitor all the huge traffic passing through the router [5]. In a military environment, these security risks can endanger sensitive monitoring assets on the network. For example, in the case of a small IP camera-based monitoring device that is cloaked secretly in a military scenario, the existence and intent of the monitoring device is clear even if only the presence or absence of a large number of encrypted streams on the network is known. If the physical location of these devices is revealed, hijacking of the other device can generate malicious and false information, which can lead to information disruption due to hijacked monitoring assets. The need for deceptive tactics is increasing owing to these conventional security methods.

In summary, deception tactics are security techniques needed to protect themselves in a way that prevents an attacker from noticing information or presence. Today’s video stream encryption methods have made significant progress; however, when sending an encrypted stream, the eavesdropping technique makes it easy to expose the device’s presence. As with encryption, the strategy of not exposing the encrypted stream to the other is increasing; therefore, the requirements for the video security method satisfying this method are as follows:

- Stealth: The sender must use an unsuspecting method that can avoid entropy scanning to protect the physical monitoring device during the transmission process.
- Confidentiality: Even if a third party acquires a stream, it should not be able to decrypt hidden images and messages.
- Computational complexity: The encryption process must be sufficiently low for application to a small monitoring device.

In this paper, our proposed method for compressed video streams satisfies all of the above conditions as follows: First, our algorithm does not use cryptographic encryption methods to satisfy stealthy and can avoid entropy scans. The method also uses deceptive cover video to be shown instead of secret video. Second, to achieve confidentiality, we analyze the header structure of video standard codecs to create a stream that is only accessed at promised devices without changing the encoder through header rules. Through this, the cover video is displayed to an unpromised device that does not know the rules of decoding a secret video.

The contribution of our research is as follows: First, we propose a new deceptive security method with the cover video that our new framework is universally applicable to five codecs. Second, the secret message space is flexible and large that the video stream can be capable. Third, since there is no hardware encoder design that can be applied as a microcircuit for small monitoring surveillance. The remainder of this paper is organized as follows. Section 2 describes the research on information security, and Section 3 describes the proposed method. Section 4 describes the implementation and evaluation of streams using the proposed method.

## 2. Related Works

**Figure 1:**
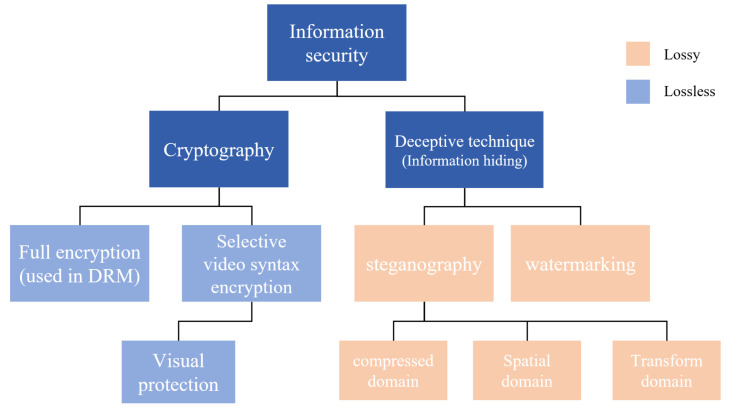
Types of information security.

The development of the Internet has enabled a vast amount of data transmission. Today, the network has huge traffic and its use is essential, but there is a problem that the data being transmitted are likely to be exposed through router. Because of this, network routers are always subject to wiretapping or malware infection. Therefore, as shown in Figure 1, the development of information security technology has been made, such as encryption method, and a deception method that does not notice whether important data are involved.

### 2.1. Selective Video Syntax Encryption

Selective video syntax encryption has been proposed as an alternative to solve the video quality degradation of compressed video steganography methods. The initial selective video syntax encryption methods began with a full stream encryption method with a symmetric key that is sometimes referred to as the naïve encryption algorithm. When establishing the standard for moving picture experts group (MPEG) video codecs, the encryption method was not specified; therefore, a symmetric key encryption method for video streams was presented via MPEG-CENC (ISO/IEC 23001-7) [6]. CENC offers a variety of encryption methods, but it recommends using AES-128bit CTR level encryption. Large video streaming providers over the Internet also use MPEG-CENC as one of the DRM methods and for delivering the key required for decryption through the RSA method [7]. In addition, the W3C, which encourages web standards, has defined encrypted media extensions to implement media DRM on HTML5 and has adopted MPEG-CENC as the standard encryption method. These initial methods have high computational complexity that syntax selective encryption methods started.

Accordingly, given that the encoding process of the video stream is already promised in a complex manner close to encryption, this method focuses on the difficulty of decryption if the decoder side does not know the changed header rules. In [8], a way to selectively apply AES encryption to only I-frames in the HEVC is proposed to alter the encryption complexity. This paper utilizes whatever I-frame is not decoded properly, and then the subsequent P-frame and B-frame images cannot obtain them as frame characteristics. However, since the motion vector information of the P-frame and B-frame remains as it is, there is a drawback in that it is possible to roughly guess the type of motion tendency video even if the video cannot be decoded. In addition, the computation cost for encryption has been reduced, but the stream length ratio occupied by the I-frame in the entire stream is still large, resulting in a high encryption cost. In [9], a random zig-zag order method was proposed using a randomly changing zigzag order of the coefficients after the DCT transform [10]. However, in this method, a block-borderline image pattern appears when decoding using a standard method without a changed order. It can be inferred that a decoding method that did not follow the rules was intentionally used. This may be seen as intentionally using a method in which inconsistency is used. In [11], a method close to encryption was achieved by transforming the binstring by transforming the context-adaptive binary arithmetic coding (CABAC) process of HEVC [12]. In the HEVC CABAC, some syntax elements are wrapped in binstring using the truncated rice code or Exp-Golomb code [13], and in this process, the binary arithmetic coding process becomes a bypass. However, the proposed process does not skip this binary arithmetic coding that easily manipulates the binstring to know them only. The CABAC process is mathematically very complex, and it is very difficult to determine the altered rule in the frequency analysis. In [14], the selective encryption was performed by complex manipulation of several syntax elements. The intra prediction mode was shifted to make distortion, and the chaotic (hash-based) encryption process was conducted for syntax elements that do not bypass the context model in the CABAC process. Nevertheless, the results of the experiment show that visual protection is weak and difficult for information security. In [15], the author proposed a method to arbitrarily mix the bytes corresponding to the NALU header using the lookup table. This achieved video encryption at a lower computational cost and allowed only those who knew the rules of the header table to decrypt it.

When sniffers look at the selective encrypted stream, it looks like a transmission of continuously intentionally corrupted video. This will allow the sniffer to know that there is a deliberate change in the video header, meaning that there is a risk that they may try to correct the header through NALU frequency analysis.

### 2.2. Compressed Video Steganography Methods

The purpose of steganography is to secretly convey messages that others do not notice on the cover stream. This deceptive method prevents a security system from noticing whether the stream is an important message [16,17]. The initial compressed video steganography research began with a video watermarking method that manipulates the lower bits of the brightness value in the raw video domain, rather than in the compressed domain, to make it visually indistinguishable. This technique has evolved by inserting an image that can be seen only in the frequency domain of the image using a simple image-processing method. Most steganography methods are based on the least significant bit (LSB) method. For example, in [18], a certain number of least significant bits are used to store a secret image of a video. Another way to hide the regularity of the LSB is to use a method of hiding by adjusting the number of LSBs between RGB channels using the eye’s sensitivity characteristics [19]. However, since it is quite easy to predict a secret message in a fixed position, this paper used a method of hiding at the adaptive region of interest (ROI) in frames [20]. Recently, steganoCNN method, using a deep learning CNN network, has also appeared [21,22,23,24].

Compared to previous studies, steganography methods in the compressed domain have more small message space. In the compressed video, the stream has a variable length and high entropy. In [25], a method of adding a 1-bit or 2-bit secret message in quantized transform coefficients for each block with a non-zero value was proposed. Here, there was an average decrease in peak signal-to-noise ratio (PSNR) of −1.38 dB due to the arbitrary modification of the quantized DCT coefficient [26] and a 4.6% increase in bitrate due to the change in run-length code length. In addition, since only a 0.2 Mbps secret message can be inserted in the video of approximately 4 Mbps, the secret video could be inserted at a 1/20 ratio. In [27], a 1-bit secret message was embedded in specific groups of the angular mode, and a threshold was presented to adjust the capacity of the secret messages. Similarly in [28], a method to add a 1-bit secret message through the most probable maximum (MPM) for each 4 × 4 luma block was proposed. This method creates an arbitrary MPM group for the two groups and if the secret message is 0 or 1, it is a structure that is mapped to each MPM group. Therefore, a wide range of predictable intra modes can be selected, meaning that the secret message is hidden while maintaining almost the same quality. However, this algorithm is only able to add 14 kB secret messages in a 200-frame sequence. Likewise, in [29,30], the MPM of the PU block was classified according to specific geopolitical to insert secret messages. In [31], the histogram shifting method was used to manipulate the highest value of intensity distribution for embedding the secret message.

Thus, conventional steganography methods in compressed space cannot add many secret messages when the secret image size is high compared to the cover image. In addition, it is difficult to flexibly adjust the length of the secret message, and intervention in the encoding process is required, so there is a drawback that the standard encoder cannot be used as it is.

Overall, there were several weaknesses in each related work. Selective encryption leaves encrypted evidence in the stream, or visual protection of secret video is not perfect. In the case of steganography, it has cover-video-quality distortion and small secret message space. Therefore, for information security, various methods should be applied simultaneously in a manner. In this paper, we propose a deception method that satisfies the requirements of conventional information security methods. Our method is faster than the selective encryption method due to header processing and has a larger secret message capacity than the steganography method.

## 3. Proposed Method

**Figure 2:**
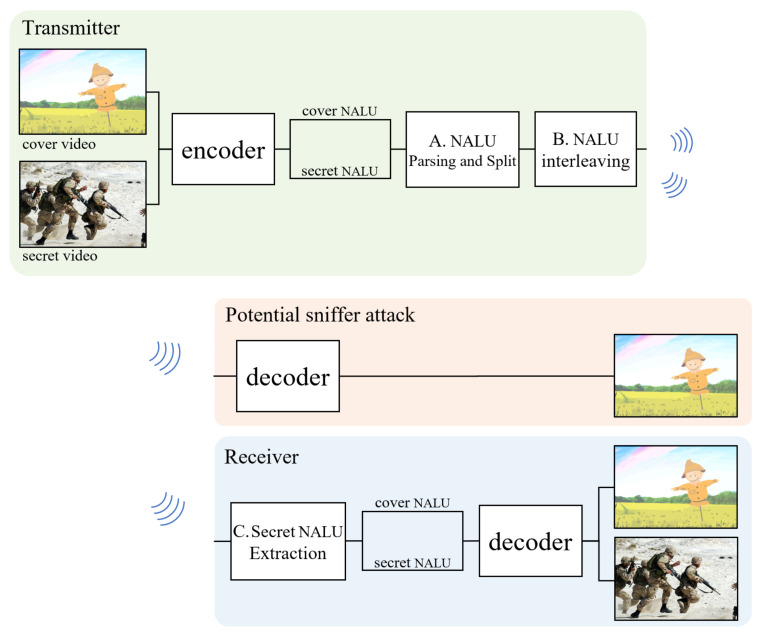
The block flow diagram of the proposed deception method.

The proposed method utilizes standard header rules for video standards, such as the selective video syntax encryption method. We use cover videos for deception, such as the steganography method. This makes it possible to reduce the calculation cost while maintaining the confidentiality of messages and the chances of a secret message being discovered. The flow of the proposed method is the same as that in Figure 2. The video transmitted over a network is transmitted not only to the receiver but also to all other hosts on the same network. Therefore, the implementation of cover video and secret video transmissions should be designed for deceptive methods. The cover video and the secret video pass through a standard encoder. When the stream is passed to an encoder, each video is converted into a series of network abstraction layers units (NALU), which can be stored as video files or transmitted to the network for a packet. Here, the secret video performs some confidential processing (e.g., encryption or byte reverse) that cannot be decrypted. Prepared cover videos and secret videos are interleaved into one stream at the A. and B. stages. The standard decoder is designed to ignore the subsequent bytes of a completely terminated NALU as a transmission error, meaning that no operation will occur on the following bytes. Since sniffing attackers do not notice any encryption or suspicious picture in both the cover video and whole stream, they do not notice the secret stream. Taking advantage of this, the deception method with a standard encoder–decoder can be implemented without the need for custom-made hardware. Most video codecs are transmitted or stored to series of NALU as video files. So the cover and secret stream can be interleaved in proposed method. Other related works also utilize the codec header, but because it is not a common codec rule like NALU, they are restricted on their type of video codec. The process of interleaving the encoded stream will be explained in more detail in the following sections, divided into A., B., and C.

*A.*  *NALU Byte Stream Parsing and Split.*

A stream of video codecs consists of various types of NALU, each NALU starting with the start code. For example, for H.264 video codecs, NALU types include SPS, PPS, and IDR-pictures. When the stream is started, the front of the NALU starts with the start code 0 × 000001. The one byte that follows the start code can know what parameter information the stream has after it, via the NALU header. SPS and PPS contain the information needed to decode the slices displayed afterwards, such as video profile, number of video intensity bits, entropy coding mode, and resolution. IDR pictures include predictive mode and Huffman-coded block data. The 1-byte NALU header includes the following three bits. First, 1-bit size forbidden_zero_bit is used to provide a notification that there is a transmission error or header violation of NALU, and it is written as 0 if NALU is normal and 1 otherwise. Next, a size of 2 bits will inform the picture reference direction of the NALU corresponding to Nal_ref_idc. In the case of the I-frame, Nal_ref_idc set 11 and in other cases of P-frame then 10, and B-frames are set as 01. The next 5-bit length nal_unit_type indicates whether the corresponding NALU contains information for decoding or picture data. To interleave NALU, the nal_unit_type is important because a different type of processing is required. Let us express 5 bits in decimals: SPS is 7, PPS is 8, IDR-picture is 5, and other types that have picture data are 1, 2, 3, and 4. Overall, three bits of forbidden_zero_bit and Nal_ref_idc in NALU type are indifferent, and the subsequent five bits are phrased only for types 1–5. Thus, after the H.264 start code, the 1-byte test pattern is shown as follows: $T_{264}:0{\mathrm{bx}}_{2}x_{1}x_{0}d_{4}d_{3}d_{2}d_{1}d_{0}$, where $x_{2}x_{1}x_{0}$ is indifferent and $d_{4}d_{3}d_{2}d_{1}d_{0}$ is 1–5 in decimal. The NALU is divided by the A. method through the test pattern and goes to the B. interleaving step.

*B.*  *NALU Interleaving.*

**Figure 3:**
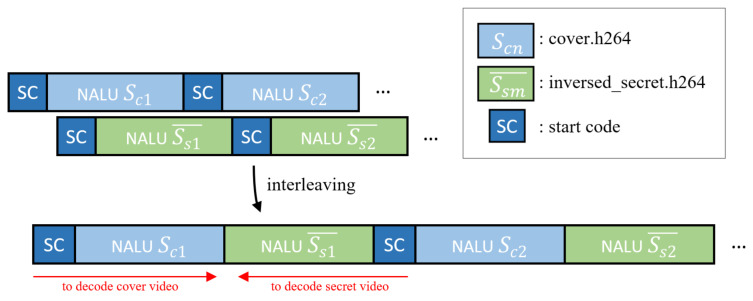
Examples of properties of interleaved NALU used in the reception method.

In this stage, the stream of the cover video and the secret video in the previous section are interleaved into one stream. Before the interleaving, there are several modulation methods of secret images for confidentiality. The modulation methods can use any invertible function, such as byte-order-reverse or encryption algorithms. However, we recommend using simple byte-order-reverse. This is because the inverse-arranged byte stream function does not increase the computational complexity and has little effect on statistical features such as entropy. Next, considering that the number of cover videos and secret NALU number are not the same, we repeat the cover NALU in the same way as the numbers of secret NALU or the trim option in interleaving. As shown in Figure 3, after the cover video is over, the stream is ignored, so we concatenate each NALU sequentially while leaving one start code. Through this process, the cover video can be obtained during forward-decoding and the secret video can be obtained in the reverse decoding direction. Let the cover video NALU of first be $S_{c1}$; the the secret video NALU of the first is $S_{s1}$. Furthermore, let this NALU, processed by a simple invertible function, be represented by $\bar{S_{s1}}$. The first stream combination $S_{1}$ that can be created at this time can be expressed as follows:

(1) $$S_{1}=\left(S_{c1},\bar{S_{s1}}\right)$$

Let the final completion stream be *S*, while *n* is the number of cover NALUs and *m* is the number of secret NALUs:

(2) $$\begin{array}{cc} S_{1} & =(S_{c1},\bar{S_{s1}})\\ S_{2} & =(S_{c2},\bar{S_{s2}})\\ \; & \;\ldots \\ S_{n} & =(S_{cn},\bar{S_{sm}}); \end{array}$$

then, the total stream *S* is

(3) $$\begin{array}{cc} 1-1S & =S_{1},S_{2},\ldots ,S_{n} \end{array}$$

where *S* refers to the entire interleaved video stream. If *n* is n<m, then, since the secret video contains more important information than the cover video, the cover video is repeatedly in a permutation loop at the first $S_{s1}$. Conversely, n>m can consider trimming the cover video.

**Figure 4:**
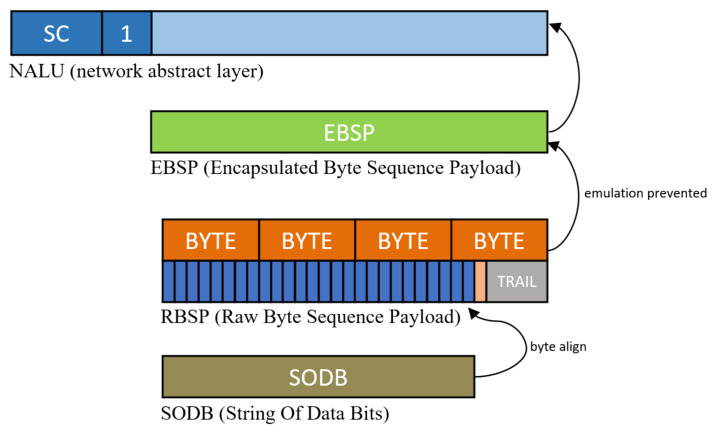
Example of NAL Unit in H.264 stream.

When interleaved with one stream, an emulation prevention process should be conducted. The secret stream space can be placed in an encrypted stream or characters, video, etc., but bytes such as start code can appear in the stream. If there is a start code in the secret video, the decoder emulates the next NALU decoding stage, meaning that an emulation prevention process must be removed. When the video encoder completes the picture in the entropy coding stage, a stream called the string of data bits (SODB) is completed, as shown in Figure 4. Each encoded symbol in the SODB is coded with a variable length with a symbol frequency; therefore, a step for aligning in byte units suitable for transmission is required. The stream for which the byte alignment is completed in the SODB is called the raw byte sequence payload (RBSP). This RBSP may occasionally contain a specific string of bytes that can be emulated. In the case of H.264, 0 × 000000, 0 × 000001, 0 × 000002, and 0 × 000003 are registered as emulation bytes. Adding emulation_prevention_three_byte 0 × 03 between the second and third bytes to prevent emulation of bytes that appear accidentally is called emulation prevention processing. After emulation prevention processing in RBSP, it is called an encapsulated byte sequence payload (EBSP), which can be stored directly in NALU. In the case of the cover NALU through the encoder, there is no need for additional emulation prevention processing because all processes are implemented in the encoder. However, the streams that processed invertible functions in the B. stage are necessary to perform the emulation prevention process again. Then, the final stream is completed by performing emulation prevention processing on the reversed stream according to the features of each codec.

*C.*  *Secret NALU Extraction.*

The secret video is returned to be decrypted at this stage for the receiver device. The secret message can be promised between transmitter and receiver in a variety of ways; if an invertible function is used for a secret message, the received stream *S* can be listed as follows.

(4) $$\begin{array}{c} S=\left(S_{c1},\bar{S_{s1}}\right),\left(S_{c2},\bar{S_{s2}}\right),\ldots ,\left(S_{cn},\bar{S_{sm}}\right) \end{array}$$

The process of extracting secret images from this stream *S* begins by dividing them into the order of NALUs received based on the start code. For example, in H.264, whenever 0 × 000001 is received in the stream, the inverse function is performed again in the whole of the previous NALU. Finally, the following stream $\bar{S}$ can be obtained.

(5) $$\begin{array}{cc} \bar{S} & =\left(\bar{S_{c1}},S_{s1}\right),\left(\bar{S_{c2}},S_{s2}\right),\ldots ,\left(\bar{S_{cn}},S_{sm}\right) \end{array}$$

With the same principle by which only the cover video was decoded in the B. stage, the decoder input $\bar{S}$ is decoded as the secret image first and the subsequent cover stream is ignored. Since the proposed method is an algorithm that combines the cover image and the secret image, image quality degradation and NALU distribution does not occur. In addition, video parameters such as quantization parameter (QP) or resolution are freely changed. Therefore, the capacity of the secret message can be set according to network bandwidth or sniffing exposure, and the statistical uniformity is the same as an original video.

## 4. Experiments and Discussions

For the experiment and evaluation of the proposed method, the proposed deception method was performed in the conformance specification for H.264 and H.265 sequences for video sequence evaluation in ITU-T [32,33]. The conformance test set is a video file that pre-encoded various parameters and image quality settings. The HEVC conformance set excluded files that did not have images with only filter data. The number of sequences in the H.264 conformance set was 135, and the number of sequences in the HEVC conformance set 142 were used. An experiment was then conducted to compare it with the conventional information security methods. The experimental environment was an Intel i5-8500 3.0 Ghz, RTX 2080 TI. The proposed deception method was conducted to evaluate the decoding availability, plausibility, encryption speed, and secret message capacity from conventional methods.

The proposed algorithm can be used in most codecs by utilizing the characteristics of NALU, but the detailed header name and byte code are slightly different for each codec, so the interleaving method used in the experiment is summarized as shown in Table 1, and the test patterns for phrases were clarified. In the case of the VP9 codec, to support the parallel decoding bundle of frames that the beginning of the picture does not start with the start code, only the byte size of the frame header is written. VP9 differs from other codecs, meaning that there is no network abstraction layer such as NALU. It has the feature that the complexity of the decoder is reduced. For this reason, it is difficult to apply the deception method to stream phrasing to the VP9 codec; therefore, it was excluded from this experiment.

Table 2 shows, decoding availability at various decoder in proposed deception methods. Both cover videos and secret videos were decodable. As described in the proposed method, the stream for the secret video can be set flexibly, but for the convenience of the experiment, the same video as the cover video was used for the secret video, and byte-reversed secret streams are used for invertible function. With the open-source S/W decoder FFMPEG, all kinds of codecs can be decoded. As a result of experimenting with the official test models JM, HM, and ITM used for standard research of video codecs, normal decoded videos are obtained. The H/W decoder used the NVDEC API provided by the NVIDIA Video Codec SDK. In the case of H.263 and IVC, H/W decoder did not exist. As cover video is decoded normally, all secret video is also decoded.

Next, to evaluate the plausibility of the stream, we performed an evaluation of HEDGE [5], which is a traffic classification algorithm that can be used in the actual sniffing method that examines how streams look similar to the original was conducted. Similarly, for the convenience of the experiment, the same cover video and secret video were used, and byte-reversed secret streams are used for invertible function in this evaluate. HEDGE performs the randomness test (NIST SP 800-22 Run test) for parts of an encrypted stream and a stream that has passed the chi square test to scan and classify common files (PDF, mp3, image, video…) [34]. HEDGE evaluates the stream by setting thresholds of χ > 99% and χ < 1% using the chi square value. High classification accuracy can be obtained if the uniformity of the distribution is checked for the length of the stream fragment size of 32 kb or more.

**Figure 5:**
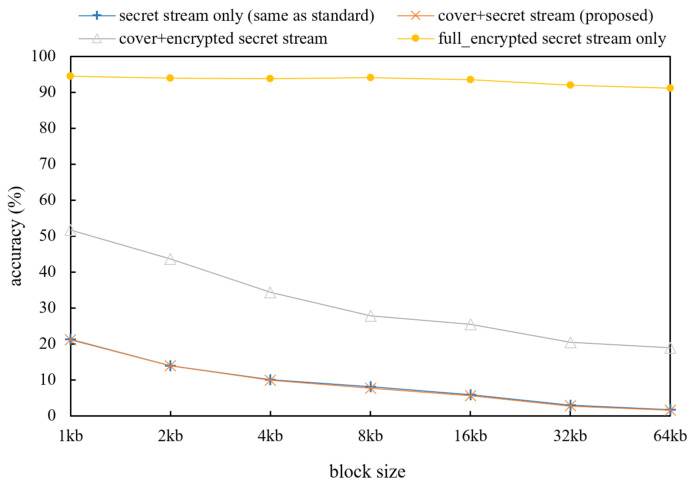
Encryption stream test results according to the HEDGE method for several interleaving versions of our system (higher accuracy means more encrypted streams).

Figure 5 shows the classification accuracy of the HEDGE algorithm for the conformance test set. The standard original video stream was difficult to distinguish from the proposed method. In the case of the original video and the proposed method, the distribution of bytes value was similar to the original because of reversed bytes of the secret video. However, the cover + full_encrypted stream shows different classification results. Since half of the stream is encrypted in this case, the probability of the encryption rate is increased in a low size block. Due to the fact that HEDGE has a sampling method, the block size shows different results.

Table 3 shows more detailed bytes’ plausibility values of the conformance set on other metric. In this table, the metric used is defined as follows:

(6) $$H=-\sum_{i=0}^{255}p_{i}log_{2}p_{i}$$

where $p_{i}$ is the expected value of the frequency of the byte value of the stream. The chi-squared method for measuring the randomness $\chi^{2}$ is defined as follows:

(7) $$\chi^{2}=\sum_{i=0}^{255}\frac{{\left(O_{i}-E_{i}\right)}^{2}}{E_{i}}$$

where $O_{i}$ is the number of observed counts of each byte value *i* and $E_{i}$ is the expected count of each byte value i. Randomness means a theoretically uniform distribution in which all $E_{i}$ values are the same. The AES-128 bit CTR recommended by the MPEG-CENC was used as the encryption method. The entropy and chi-square values were compared for the four previous evaluations. The cover + secret stream indicated that the entropy and chi-square values were also approximately the same as the original. Similarly, the confidential stream shows a high chi-square value that can be revealed in the encrypted stream detection technique. Furthermore, there was a slight increase in the chi square value in cover + secret stream, it appears that the start code, which corresponds to the start of NALU, faces the cover video and the secret video at the start code point. Through the evaluation, the proposed deception method shows similar results to the standard video stream.

The following is the encryption speed evaluation of the proposed method with related encryption works. Table 4 shows the complexity of comparing the coding time of the proposed method with the conventional header encrypt method [15] and full stream encryption [7]. For more valuable evaluation, File I/O speed for uniformity of experiment is excluded. We measured the encryption speed with all streams loaded into memory. The average time saving (ATS) is defined as follows:

(8) $$ATS\left(\%\right)=\frac{Enc.time(anchor)-Enc.time(proposed)}{Enc.time(anchor)}\times 100$$

It can be seen that the cover + secret method has more bytes to be processed, but the operation speed is much faster. The full_encryption method increased the bytes processing time, but the proposed deception method increased the computational complexity according to the number of NALU processed. Since HEVC sequences have few NALU, the average time saving tends to relatively improve.

Next, the capacity compared with the related steganography is as follows. The proposed method can flexibly control the cover video to contain the secret video for which we used a 1:1 ratio for this capacity comparison. However, other steganography studies have generated a large amount of overhead such as that seen in Table 5. To get other steganography methods with maximum capacity, all intra profile was used with QP 32 for [29] in class 1, 2, and 3. Furthermore, the baseline profile of QP 22 was used for [31] at a 50% embedding rate.

The other comparison methods are only a few secret messages of 0.33% and 2.72% in the cover video. Therefore, assuming that the same secret message is required, the required overheads are close to 302 times and 36 times, respectively.

Finally, Table 6 shows a comparison with other information security methods; the first, fourth rows are representative steganography methods, and the fifth and sixth rows are selective video syntax encryption methods. The steganography method was difficult to use for the deception method because there was not enough secret message space to hide video, and the selective video syntax encryption method was difficult to use as a deception method because of the absence of a cover video. However, the proposed method showed advantages in terms of capacity and existence of deceptive cover video.

Overall, a comparison of the other features from related works and the proposed method is presented as follows. 

Comparison of selective video syntax encryption method: Selective video encryption techniques typically output visually protected hard identification images or non-decodable image. This makes the sniffers aware that the stream is confidential. However, the proposed method decoded cover video first that was not classified as an encrypted stream, as shown in experiments. However, the proposed deception method utilizes the standard NALU structure such that our deception methods have the same tolerance as the standards video codec, even if the NALU is damaged. The data after excluding the damaged NAL can be decoded. Therefore, the proposed method is effective against data contamination. 

Comparison with the compressed video steganography method: Compressed video streams have very high entropy, so it is difficult to insert large secret messages in the compressed domain. For this reason, the conventional state-of-the-art compressed video steganography method has a very small secret message space. Considering that the cover video and secret message have an approximately 20:1 ratio, only a few secret messages can be transferred. Furthermore, the steganography method manipulates the symbol during the encoding process so that it is necessary to change the hardware design of the encoder. Therefore, there is a disadvantage that the hardware-implemented video encoder cannot be used as the original design. Furthermore, because the secret message manipulates the video symbol of the cover video such that it causes a unique pattern and suspicion in the cover video. On the other hand, the proposed method can freely adjust the ratio between the cover video and the secret video and can adjust the video quality of the secret video according to the purpose of flexibly dealing with it. Furthermore, there is an advantage that the hardware designed standard encoder can be used as the original, and the video quality of the cover video is not distorted in the proposed method. 

## 5. Conclusions

In this paper, we proposed a new compressed video stream deception method that can transmit a secret video in a cover video using the header structure of a video codec. The results show that the proposed deception method is almost the same as the original stream that avoided network scan eavesdropping for a network traffic analysis method HEDGE and reduced the computational complexity compared to encryption methods with partial NALU processing. Our deceptive method has a large secret space compared to other steganography methods, which was appropriate for video deception techniques. In addition, our proposed method uses an original video encoder design that can be applied to small monitoring devices in real-time with a standard. Therefore, compared to conventional encryption and steganography methods, it was confirmed that there are advantages in applying a small device. According to this study, it is expected that information security for deceptive methods will be diversified.

## Figures and Tables

**Table 1:** Test pattern for the NALU parsing process of the proposed method.

Codecs	Interleaving Point	Test Pattern	
H.263	Picture start code	0b0000 0000 0000 0000 1000 00	all point of start codes
MPEG-2	slice header	0b0000 0000 0000 0000 0000 0001 hhhh hhhh	h: 01-AF as hexadecimal
H.264	IDR, non-IDR, slice	0b0000 0000 0000 0000 0000 0001 xxxd dddd	x: do not-care, d: 1-5 as decimal
HEVC	All of coded slice	0b0000 0000 0000 0000 0000 0001 xddd dddx	x: do not-care, d: 0-9, 16-21 as decimal
IVC	I-frame, PB-frame	0b0000 0000 0000 0000 0000 0001 hhhh hhhh	h: B3, B6 as hexadecimal
VP9	-	-	-

**Table 2:** Decoding possibility according to codec type.

Test Codecs	S/W Decoder	H/W Decoder
H.263	FFMPEG 4.3.2	-
MPEG-2	FFMPEG 4.3.2	NVDEC
H.264	JM v19.0	NVDEC
HEVC	HM v16.17	NVDEC
IVC	ITM v14.1	-

**Table 3:** Comparison of plausibility of several versions.

Interleaving Methods	Entropy	Chi-Square Value
secret stream only	7.9445	9975.5
cover + secret stream	7.9440	10,856.5
cover + encrypted secret stream	7.9620	6082.5
full_encrypted secret stream only	7.9969	255.7

**Table 4:** Comparison of computational complexity with selective video syntax encryption methods.

Sequence	Header Encrypt [15]		ATS(%)	Full_Encryption [7]		ATS(%)	Proposed Method	
	Bytes	Time		Bytes	Time		Bytes	Time
H.264 conformance set	-	-	-	474,389	0.0056 s	40.0	948,735	0.0034 s
HEVC conformance set	397,590	0.0026 s	69.0	397,590	0.0049 s	83.8	795,139	0.0008 s

**Table 5:** Comparison of required cover video sizes with the same secret video size.

	Required	Total Size (kb)	Secret Message	
	Overhead Ratio		Capacity (kb)	Ratio (%)
intra position [29]	302.94	97,324	271.99	0.33
histogram [31]	36.63	11,986	356.83	2.72
proposed method	1.00	49,561	49,561	100

**Table 6:** Feature compare with conventional methods.

	Cover Video Distortion	Secret Payload	Visual Protection of Secret Video	Available Video Codec
non−zero [25]	−1.38 dB	20.19 bits/MB	−	H.264
MPM hiding [28]	−0.04 dB	64.6 bits/frame	−	H.264
angular pos [29]	−0.97 dB	59.84 bits/frame	−	HEVC
histogram [31]	−1.30 dB	423.02 bits/frame	−	H.264
header encrypt [15]	no cover video	−	◯	HEVC
BAC skip [11]	no cover video	−	△	HEVC
Proposed method	0.00 dB	flexible	◯	H.263, MPEG-2, H.264, HEVC, IVC

◯: Completely undecoded or protected by the cover video; △: A spatial correlation between blocks shown or block movement presence.

## Data Availability

Not applicable.

## Abbreviations

The following abbreviations are used in this manuscript:

MPEG	Moving Picture Experts Group
DASH	Dynamic Adaptive Streaming Over HTTP
DRM	Digital Rights Management
W3C	World Wide Web Consortium
IoT	Internet of Things
LAN	Local Area Network
DCT	Discrete Cosine Transform
PSNR	Peak Signal-to-Noise Ratio
MPM	Most Probable Mode
AES	Advanced Encryption Standard
DES	Data Encryption Standard
AVC	Advanced Video Coding
HEVC	High Efficiency Video Coding
IVC	Internet Video Coding
VP9	Video Predictor 9
CABAC	Context-Adaptive Binary Arithmetic Coding
NALU	Network Abstraction Layer Unit
SPS	Sequence Parameter Set
PPS	Picture Parameter Set
IDR	Instantaneous Decoder Refresh
SODB	String of Data Bits
RBSP	Raw Byte Sequence Payload
EBSP	Encapsulate Byte Sequence Payload
FFMPEG	Fast Forward MPEG
NVDEC	Nvidia Video Decoder
NIST	National Institute of Standards and Technology
SP 800-22A	Special Publication 800-22 Revision 1A
